# Automated Detection of Epileptic Biomarkers in Resting-State Interictal MEG Data

**DOI:** 10.3389/fninf.2017.00043

**Published:** 2017-06-30

**Authors:** Miguel C. Soriano, Guiomar Niso, Jillian Clements, Silvia Ortín, Sira Carrasco, María Gudín, Claudio R. Mirasso, Ernesto Pereda

**Affiliations:** ^1^Instituto de Física Interdisciplinar y Sistemas Complejos, Consejo Superior de Investigaciones Científicas (CSIC), Campus Universitat Illes BalearsPalma de Mallorca, Spain; ^2^McConnell Brain Imaging Center, Montreal Neurological Institute, McGill UniversityMontreal, QC, Canada; ^3^Laboratory of Cognitive and Computational Neuroscience, Center of Biomedical Technology, Politechnical University of MadridMadrid, Spain; ^4^Department of Electrical and Computer Engineering, Duke UniversityDurham, NC, United States; ^5^Teaching General Hospital of Ciudad RealCiudad Real, Spain; ^6^Electrical Engineering and Bioengineering Group, Department of Industrial Engineering, Instituto Universitario de Neurociencia, Universidad de La LagunaTenerife, Spain

**Keywords:** epilepsy, magnetoencephalography, randomized neural networks, automated classification

## Abstract

Certain differences between brain networks of healthy and epilectic subjects have been reported even during the interictal activity, in which no epileptic seizures occur. Here, magnetoencephalography (MEG) data recorded in the resting state is used to discriminate between healthy subjects and patients with either idiopathic generalized epilepsy or frontal focal epilepsy. Signal features extracted from interictal periods without any epileptiform activity are used to train a machine learning algorithm to draw a diagnosis. This is potentially relevant to patients without frequent or easily detectable spikes. To analyze the data, we use an up-to-date machine learning algorithm and explore the benefits of including different features obtained from the MEG data as inputs to the algorithm. We find that the relative power spectral density of the MEG time-series is sufficient to distinguish between healthy and epileptic subjects with a high prediction accuracy. We also find that a combination of features such as the phase-locked value and the relative power spectral density allow to discriminate generalized and focal epilepsy, when these features are calculated over a filtered version of the signals in certain frequency bands. Machine learning algorithms are currently being applied to the analysis and classification of brain signals. It is, however, less evident to identify the proper features of these signals that are prone to be used in such machine learning algorithms. Here, we evaluate the influence of the input feature selection on a clinical scenario to distinguish between healthy and epileptic subjects. Our results indicate that such distinction is possible with a high accuracy (86%), allowing the discrimination between idiopathic generalized and frontal focal epilepsy types.

## 1. Introduction

Epilepsy is defined as a neurological disorder associated with seemingly random occurrences of recurrent seizures. It is related to a decrease in quality of life, with mortality rates 2–3 times higher for epileptic patients than for the general population (Bell et al., 2001). Epilepsy affects approximately 1% of world's population, one third of which are resistant to pharmacological treatment (Pitkänen et al., 2016). Although, not in all cases, most of these patients could benefit from a variety of surgical options instead.

There are different types of epilepsy (ILAE, 1989). In many cases, epileptic seizures originate in a single brain area, called the focus, which acts as the trigger of the abnormal brain functioning. In this case, we talk about focal epilepsy. In other cases, however, the brain activity of the patients look typically normal, without any apparent structural brain abnormality. This type of epilepsy, which is believed to have a strong underlying genetic basis, is known as idiopathic generalized epilepsy.

A crucial step to improve the quality of life of epileptic patients is an early diagnosis so they can receive the appropriate treatments as fast as possible. Efforts to ascertain newly diagnosed cases of epilepsy are particularly challenging. Clinical diagnosis can be a complex process, as no specific biologic markers exist and seizures are associated with a wide range of disease conditions. Clinically, epilepsy is normally studied with a series of tests, which include neuroimaging and neurophysiological recordings. In the latter case, scalp EEG is often the preferred choice, because, despite its relatively poor spatial resolution, it allows, e.g., for the localization of the epileptic focus non-invasively. Yet in the case of pharmacologically intractable epilepsy, intracranial EEG recordings (electrocorticograms, ECoG) are necessary for a more precise localization (and even, functional characterization) of the focus. For the evaluation of epilepsy, meta-analysis studies have found wide variability in incidence estimates and in the quality of results, depending on the classifications one uses for epilepsy and epileptic seizures (Kotsopoulos et al., 2002).

The development of computer aided techniques to detect epilepsy can help to improve the health care and quality of life of epileptic patients. Several machine learning techniques have been applied to enhance the epileptic detection. For instance, in Holden et al. (2005), an algorithm based on conditional logistic regression examining combinations of diagnosis, diagnostic procedures, and medication classified 90% of the epileptic cases. Machine learning algorithms have also been used to distinguish between interictal or preictal segments to forecast epileptic seizures (Park et al., 2011; Brinkmann et al., 2016; Myers et al., 2016) or to estimate the seizure onset times (Chan et al., 2008). Most of these algorithms use information from the EEG such as the power spectra, correlation between EEG sensors, signal variance, and/or phase synchronization in order to achieve high prediction accuracy (Chan et al., 2008; Park et al., 2011; Brinkmann et al., 2016; Myers et al., 2016).

In this work, we have developed a machine learning algorithm that classifies interictal brain activity segments recorded with magnetoencephalography (MEG) belonging to either epileptic patients (frontal focal and idiopathic generalized) or healthy subjects. To that end, we compute a reduced subset of features from the interictal brain activity (closed eyes) recorded with MEG that provide a comprehensive description of the brain activity. These features are obtained from either the computation of two bivariate indices of phase synchronization at the sensor level or a more traditional power spectral analysis of the MEG data, in which all eptileptiform activity has been removed.

Recently, MEG has shown to be a very useful tool to assess epileptic activity (Almubarak et al., 2014; Englot et al., 2015; Niso et al., 2015; Chen et al., 2016; Hillebrand et al., 2016; Murakami et al., 2016; Nissen et al., 2016). Interestingly, MEG can be used as a capable surrogate for EEG/ECoG to identify the location of the focus from epileptiform discharges, with higher sensitivity than EEG (Hunold et al., 2016) and even comparably to the ECoG while being much less invasive (Almubarak et al., 2014; Murakami et al., 2016). Equally important is the fact that MEG also allows studying the differences in interictal brain activity without epileptiform discharges on epileptic patients as compared with healthy subjects (Englot et al., 2015; Niso et al., 2015). Specifically, the application of functional connectivity methods to resting state MEG data has disclosed the existence of distinctive functional connectivity patterns in the epileptic brain (see e.g., Englot et al., 2015, 2016; Niso et al., 2015 and references therein). These results pave the way to use resting state interictal MEG data as a biomarker for epilepsy.

To carry out the analysis, we apply a conceptually simple machine learning algorithm based on random mappings, known as extreme learning machine (ELM) (Schmidt et al., 1992; Pao et al., 1994; Huang et al., 2006). ELM relies on a feedforward neural network with a single hidden layer, whose connection weights are initialized with random values and only the output weights are optimized. The conceptual simplicity of ELM makes hardware implementations of this algorithm possible in electronics (Decherchi et al., 2012; Frances-Villora et al., 2016) and optics (Ortín et al., 2015). Here, we use the algorithm to classify the subjects into healthy or epileptic (idiopathic generalized epilepsy or frontal focal epilepsy) and explore the benefits of using different features obtained from the MEG data as inputs to the algorithm.

In contrast to previous studies, we here focus on the identification of epileptic biomarkers in resting-state interictal brain activity. We seek to elucidate whether epileptic brains behave differently than normal ones even in the absence of seizures or any other epileptiform activity. Our study is not only interesting from a methodological point of view but potentially also from a clinical perspective, since there are patients who have no obvious or frequent spikes. This may lead to an incorrect diagnosis as having non-epileptic events when in fact they do have epilepsy. In these cases, an algorithm such as the one presented here could suggest the need for a closer examination and possible new diagnosis.

## 2. Materials and methods

**Figure 1:**
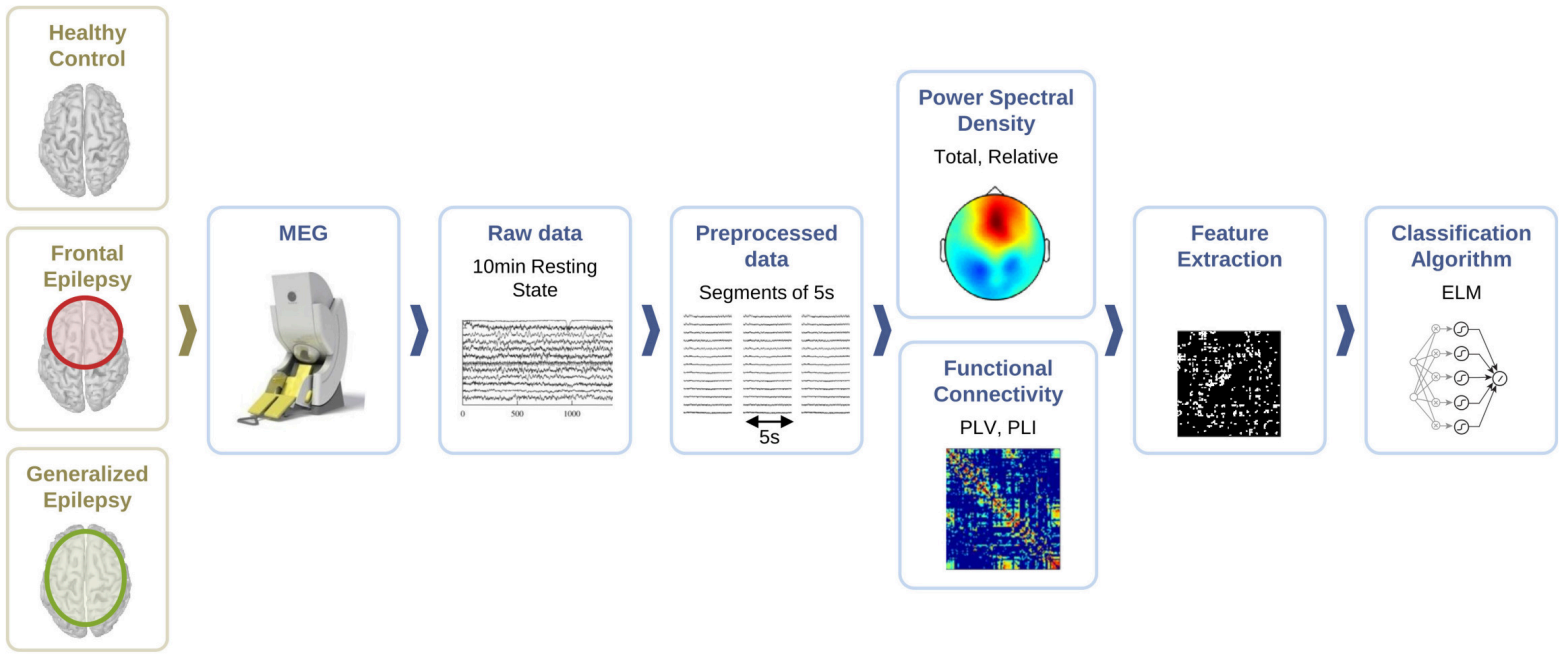
Schematic representation of the procedure followed for the automated detection of generalized and focal epilepsy on the resting state interictal MEG.

For the classification of healthy and epileptic subjects, we follow a systematic approach in successive steps. First, we acquire the MEG recordings of all subjects. The information of the participants in this clinical study is detailed in Section 2.1. Second, we pre-process the MEG recordings to extract several temporal segments of high signal quality for each subject. Third, we calculate four different features for each MEG segment, namely the total and the relative power spectral densities, the phase-locked value and the phase-lag index. Using these features, we first classify the segments using the ELM algorithm. Subsequently, we classify the subjects according to the majority class of each subject segments'. The complete procedure is schematically summarized in Figure 1 and each step of the process is detailed in Sections 2.2–2.6.

### 2.1. Participants

For the current clinical study, we analyzed 42 subjects: 14 patients (9 male) suffering from frontal focal epilepsy, 14 patients (5 male) suffering from idiopathic generalized epilepsy (they all meet the criteria Juvenile Myoclonic Epilepsy or presumed genetic, as the new terminology stands; ILAE, 1989), and 14 (7 males) healthy subjects. The demographics of the subjects is summarized in Table 1.

**Table 1:** Demographics of the subjects that volunteered to the current clinical study.

	**Focal epilepsy**	**Generalized epilepsy**	**Healthy**
Number of subjects	14	14	14
Average age and standard deviation	36 ± 16 years	28 ± 7 years	20 ± 4 years

All patients were classified independently by two neurologists. For the diagnosis and classification of seizures and epileptic syndromes, the clinical and electroencephalographic classifications of the ILAE were applied (ILAE, 1989). Patients were seen consecutively during the period between May 2009 and December 2011 in the outpatient clinic of neurology at the University General Hospital of Ciudad Real, with an average evolution track of 10 years, and were free from epileptic crisis during the last 6 months prior to the recording. They were all free from mental retardation, connate anoxia history, history of head trauma or meningitis in early childhood. Each clinical history gathered epidemiological data (sex, age of onset of the first seizure, personal history, pregnancy, and delivery), clinical data (type of seizures and neurological findings), and treatments used. These data were combined with additional examinations [EEG, neuroimaging studies, magnetic resonance imaging (MRI) of 1.5 or 3 Tesla following the protocol for epilepsy that includes 3D-T1 isotropic voxel sequences of 1 and 2 mm axial and coronal slices with T2, FLAIR, and T2^*^ sequences], to establish if the etiology was idiopathic, symptomatic, or cryptogenic. The entire cohort of patients was also evaluated using a comprehensive neuropsychological test battery. The patients took a range of medications with different mechanisms of action. In this study, patients were chosen in such a way that the medication was similar and controlled between both epilepsy groups (Niso et al., 2015). All data were analyzed anonymously and ethical approval was granted by the Local Ethics Committee of the Teaching General Hospital of Ciudad Real.

### 2.2. MEG recordings and data pre-processing

For all subjects, we recorded brain activity during resting state with MEG at the Center for Biomedical Technology, CTB, Madrid. MEG recordings were obtained using a 306-channel whole head Elekta Neuromag MEG system (Elekta Oy, Helsinki, Finland), comprising 102 magnetometers and 204 planar gradiometers in a helmet-shaped array covering the entire scalp, while subjects were seated inside a magnetically shielded room (Vacuumschmelze GmbH, Hanau, Germany). Eye movements were monitored by simultaneously recording the electrooculogram with three Ag/Cl electrodes, two above and below the right eye and one at the right earlobe (ground reference). Four head position indicator (HPI) coils, whose data were used to correct head movement during the session, were placed on the scalp, appropriately spaced in the region covered by the MEG helmet. The locations of the nasion, two pre-auricular points, and the 4 HPI coils were digitized prior to each MEG study using a 3D-digitizer (FASTRACK; Polhemus, Colchester, VT) to define the subject-specific Cartesian head coordinate system. One hundred to two hundred additional anatomical points were digitized on the head surface to provide a more accurate shape of the subject's head.

MEG data were acquired (sampling rate of 1 kHz, on-line band-pass filter ranging from 0.10 to 330 Hz) during a single session of resting state (Niso et al., 2015). For each session, we analyzed data obtained during 10 min with eyes closed. These data were free from blinking artifacts and interictal epileptiform discharges. External noise was removed, as recently suggested in MEG literature (Gross et al., 2013), by using the temporal extension of Signal-Space Separation (tSSS) (Taulu et al., 2005) implemented with the MaxFilter software (version 2.0 ElektaNeuromag; sliding window of 10 s, subspace correlation limit of 0.98; Hillebrand et al., 2013), and configured with an inner expansion order of 8. We also applied movement compensation to our data, to correct for subject's head movement during the recording. Spikes and spike-wave complexes were observed in the data but were removed from the analysis since our aim is to classify subjects during the absence of epileptiform activity. This is a key aspect of our study since it is not always easy to record epileptiform activity during short periods of recording times. Following this procedure, and within the 10 min with eyes closed, we selected the 40 most stationary segments of 5 s length for each patient by using the Kwiatkowski-Phillips-Schmidt-Shin (KPSS) test for stationarity (Kipiński et al., 2011).

For each temporal segment, we consider the information of the 102 magnetometers in the MEG system, i.e., each segment of 5 s contains information of 102 different channels.

### 2.3. Feature extraction

We compute four different features for each segment, namely the total and relative power spectral densities (PSD), the phase-locking value and the phase-lag index, described below. These features are calculated for every segment and sensor (magnetometer) of each subject. All these measures are calculated for a range of frequencies of interest in the interval (4–40) Hz, in steps of 2 Hz. As a result, the computation of each feature leads to a matrix of dimensions 102 x 18 (sensors x frequency intervals), which is used as input to the machine learning algorithm discussed in Section 2.4 for further classification of the MEG segments and subjects.

**Figure 2:**
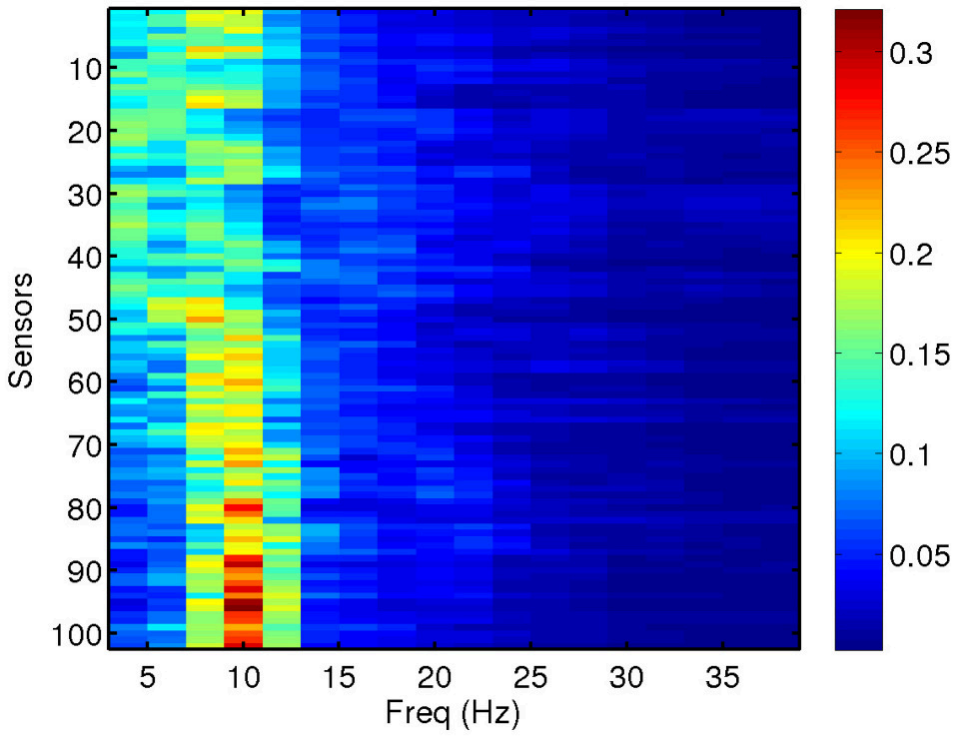
Relative PSD for a single MEG segment of a given subject in this study.

We compute total and relative PSD of the MEG data (40 segments for each patient) for the range of frequencies of interest. Relative PSD refers to the total PSD on each sensor on a certain frequency band divided by the total PSD on that sensor. In Figure 2, we show an example of the relative PSD for one MEG segment of a given subject. This image contains the spectra of the 102 sensors (y-axis) in 18 frequency intervals of 2 Hz (x-axis).

We analyze synchronization and functional brain connectivity from the MEG data by computing two bivariate phase synchronization measures: the phase-locking value (PLV) (Lachaux et al., 1999; Mormann, 2000) and the phase-lag index (PLI) (Stam et al., 2007). The use of PLV and PLI as features is motivated by the fact that epilepsy has been historically seen as a functional brain disorder associated with excessive synchronization of large neuronal populations (Jiruska et al., 2013). Increasing evidence shows that epileptiform phenomena, particularly seizures, follow a characteristic dynamical evolution of synchronization. Desynchronization is often observed preceding seizures while high levels of synchronization are observed toward the end of seizures. For computations, we use the HERMES toolbox (Niso et al., 2013) (freely available at http://hermes.ctb.upm.es/). Amplitude-based measures such as mutual information or measures of generalized synchronization are less suitable to analyze synchronization regimes in which the amplitudes remain only weakly correlated. Instead, PLV is able to detect weak synchronization regimes, where the phases of the oscillatory component are coupled but the amplitude may not be (Rosenblum et al., 1996; Hramov et al., 2005). In turn, PLI is a measure of asymmetry for the distribution of phase differences between two signals (Stam et al., 2007). PLV is sensitive to both zero and non-zero lag synchronization, while PLI is only sensitive to the latter one. Zero lag synchronization is usually regarded as the result of the same neural source being picked up by two different sensors. In addition, zero lag synchronization can be achieved if two neural sources are indirectly connected through a third one, acting as a dynamical relay (Vicente et al., 2008), which would be overlooked should we only focus on PLI. Therefore, we include both PLV and PLI as features in our study so that we can elucidate whether information from only direct connections (PLI) is enough for a good discrimination or, instead, including information on both direct and zero lag functional connectivity is best for this purpose.

**Figure 3:**
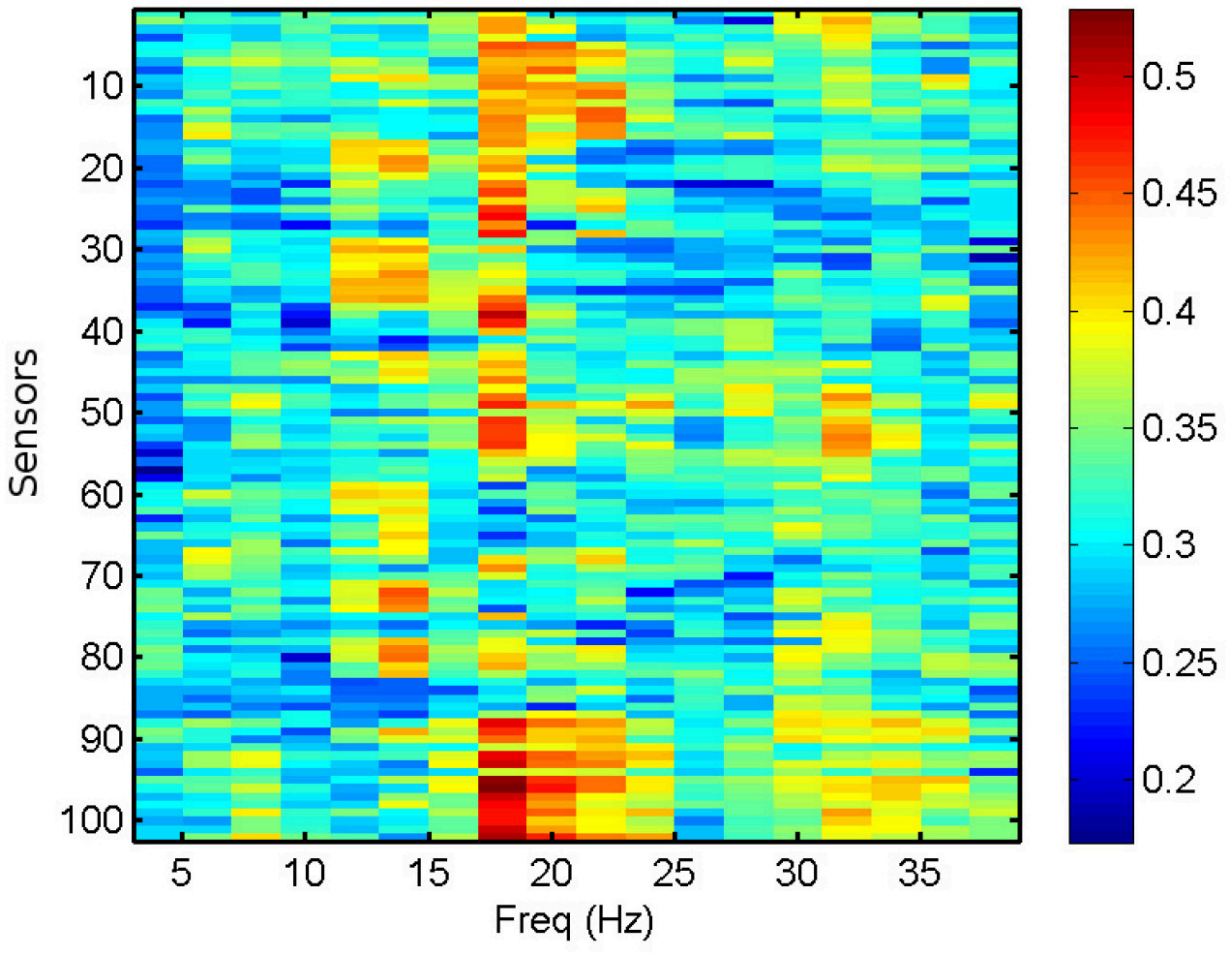
Average PLV per sensor for a single MEG segment of a healthy subject.

We compute the PLV and PLI on bands centered in the frequencies ranging from 4 to 40 Hz with a resolution of 2 Hz, which represents a reasonable trade-off between two mutually opposed requirements. First, the frequency intervals should be narrow enough to allow for proper phase reconstruction (Thiel et al., 2006). Second, selecting extremely narrow intervals would require a very high-order filter, which may distort part of the data segments due to border effects. It is common practice to apply a threshold of significance to the values of PLV and PLI (Kim et al., 2013). Here, we keep the 40% (50%) most significant values of PLV (PLI) and zero the rest, keeping a fixed proportion of the highest links. However, we have checked that the results do not change qualitatively by choosing other thresholding levels. We use the average PLV and PLI as estimators of the functional connectivity at each sensor location, within the frequency bands mentioned above. PLV and PLI are computed for each pair of sensors, such that the PLV_*i, j*_ (PLI_*i, j*_) is the PLV (PLI) between sensors *i* and *j*. The corresponding average PLV (PLI) of sensor *i* is then the average of the PLV_*i, j*_ (PLI_*i, j*_) sum over all *j*, with *i* ≠ *j*. In Figure 3, we show the computed average PLV for a single MEG segment of a given subject. Similarly to the procedure described for the total and relative PSD, the results of the PLV or the PLI are passed in matrix format of dimensions 102 x 18 (sensors x frequency intervals) as input to the machine learning algorithm for the classification of the MEG segments and subjects.

### 2.4. Classification algorithm (ELM)

Here, we use randomized neural networks as the classification algorithm. In particular, we employ ELMs, which are supervised machine learning models based on a random mapping of the inputs into the high dimensional space of the neural network. ELMs aim at learning the conditional expected value of a m-dimensional output **y**(*n*) as a function of a d-dimensional input **x**(*n*), based on a number of examples *n* = 1, …, N. The particularity of this approach is the use of an intermediate hidden layer with *D* neurons, where inputs are randomly mapped into a nonlinear form, yielding a new, transformed *D*-dimensional **r**(*n*) space. The elements of the hidden layer are often denoted as neurons since these approaches were initially developed in the framework of neural networks.

The ELM model can be written in the following general form (Huang et al., 2006; Ortín et al., 2015):

(1) $$\begin{array}{l} \text{r}\left(n\right)=F\left(\eta {\text{W}}_{in}\text{x}\left(n\right)\right), \end{array}$$

(2) $$\begin{array}{l} \text{o}\left(n\right)={\text{W}}_{out}\text{r}\left(n\right), \end{array}$$

where **r**(*n*) and **o**(*n*) are the states of the hidden layer neurons and the predicted output values, respectively. **W**_*in*_ is the *d* × *D* random input weight matrix that maps the input (dimension *d*) onto the hidden layer (dimension *D*), *F* is the hidden layer nonlinear activation function, and η is an input scaling factor. We use *F*(*z*) = *sin*^2^(*z* + ϕ), where *z* is the mapped input signal, as the nonlinear activation function. For this activation function, the operating point and hence the degree of nonlinearity can be adapted by varying the phase ϕ. Machine learning models with this nonlinear function can be easily implemented in optoelectronic hardware, allowing for ultra-fast processing speeds (Larger et al., 2012; Soriano et al., 2015a).

Learning from data is efficiently achieved through the random mapping technique, since the only weights trained in this approach are those corresponding to the output connections, **W**_*out*_. **W**_*out*_ are usually computed by minimizing the squared error between the real **y**(*n*) and the predicted **o**(*n*) output values. In our case, we have used a simple linear regression (Huang et al., 2006). The input data **x**(*n*) are first multiplied by **W**_*in*_ and then normalized to zero mean and unity standard deviation. In our study, the input data correspond to the flattened matrices of the computed features. Thus, the input features for each segment typically have a dimension *d* = 1, 836 (102 x 18).

The values of **W**_*in*_ are continuous, drawn from a uniform distribution over the interval [1, −1]. Since they are randomly chosen, the performance of a single realization of the algorithm might depend on the particular values of these random input weights. To avoid this uncertainty, we use an ensemble classifier that produces statistically robust results. This ensemble classifier is a combination of the results from a pool of independent realizations of **W**_*in*_. Here, we use ensemble classifiers that combine the results of 100 different realizations using a simple majority rule.

One of the main advantages of this classification algorithm based on random mappings is that the parameters of the algorithm can be easily retrained if new data is incorporated, either from new subjects or more measurements from the same subjects. This property is particularly suited for applications where data is acquired sequentially, e.g., new subjects being incorporated to the study.

### 2.5. Performance evaluation

The performance of the classifiers is measured in terms of four quantities defined as the number of true positives (TP), the number of true negatives (TN), the number of false negatives (FN), and the number of false positives (FP). These quantities are often displayed as a confusion matrix, following a table layout. As shown in Table 2, the columns of the confusion matrix represent the instances in a predicted class while the rows represent the instances in a true class.

**Table 2:** Confusion matrix for a binary classifier.

		**Prediction**	
		**Control**	**Pathological**
True condition	Control	TN	FP
	Pathological	FN	TP

*TN, FP, FN, and TP stand for true negatives, false positives, false negatives, and true positives, respectively*.

From the confusion matrix, it is easy to compute the total proportion of correct predictions (accuracy), the percentage of positives that are correctly identified as having the condition (sensitivity), and the percentage of healthy subjects that are correctly identified as not having the condition (specificity), defined as follows:

(3) $$\begin{array}{l} Accuracy\;=\;\frac{TP+TN}{TP+TN+FP+FN}\;, \end{array}$$

(4) $$\begin{array}{l} Sensitivity\;=\;\frac{TP}{TP+FN}\;, \end{array}$$

(5) $$\begin{array}{l} Specificity\;=\;\frac{TN}{FP+TN}\;. \end{array}$$

At the subject level, the results are presented in the form of the confusion matrix. At the segment level, we make use of the receiver operating characteristic (ROC) curves to present the results. In statistics, the ROC curve is used as a graphical tool to illustrate the performance of a binary classifier as its discrimination threshold is varied. The ROC curve is created by plotting the probability of detection (sensitivity) against the probability of false alarm (1-specificity) at various threshold settings. Thus, each point on the ROC curve represents a sensitivity/specificity pair corresponding to a given discrimination threshold. Performance is measured by the area under the ROC curve. This area under the curve (AUC) measures the ability of the classifier to correctly identify those subjects with and without the epileptic condition. An area of 1 represents a perfect classifier, while an area of 0.5 represents the same classification as in a random guess.

### 2.6. Algorithm training and parameter optimization

For the training and test of the ELM classifiers, we follow a leave-one-out cross-validation procedure. This procedure is a standard way to evaluate the performance of machine learning algorithms, especially when the number of subjects is small as in our case. Leave-one-out cross-validation involves using one subject as the test set for the classifier trained using the remaining subjects. In our case, we have 14 subjects for each condition (healthy, focal, generalized) that we divide in 14-folds. In this manner, one subject of each condition is tested in every fold and all subjects are tested exactly once after the leave-one-out cross-validation. In this way, we do not overestimate the accuracy of the algorithm using intra-patient information to classify the segments. The subject-wise approach, in contrast to a segment-wise one, estimates better the accuracy of the algorithm (Saeb et al., 2016).

The ELM classification algorithm is first trained to classify MEG segments using as input one or more of the features defined in Section 2.3. After the training and for each fold, the algorithm assigns a condition to each segment of the test subject. It then decides on the condition of the test subject by using a majority rule over the results for the segments of this test subject. This process is repeated for each of the 14 test folds and the final accuracy of the classifier is obtained by averaging over the individual fold accuracies. We note that the training and test sets for each fold contain records from non-overlapping subjects since our goal is to build a classifier that can generalize to identify epilepsy traits rather than the subject itself.

The parameters of the ELM algorithm are optimized for the identification of epileptic and healthy segments. Since the current study is performed on a total of 42 subjects, with 40 temporal segments each, this amounts to 1,680 (42 x 40) examples. This reduced amount of training examples limits the number of neurons that can be used in the hidden layer of the ELM, which must be much smaller than the number of examples. In this work, we present results for a hidden layer with *D* = 350 neurons since we have checked that it gives a consistent performance.

**Figure 4:**
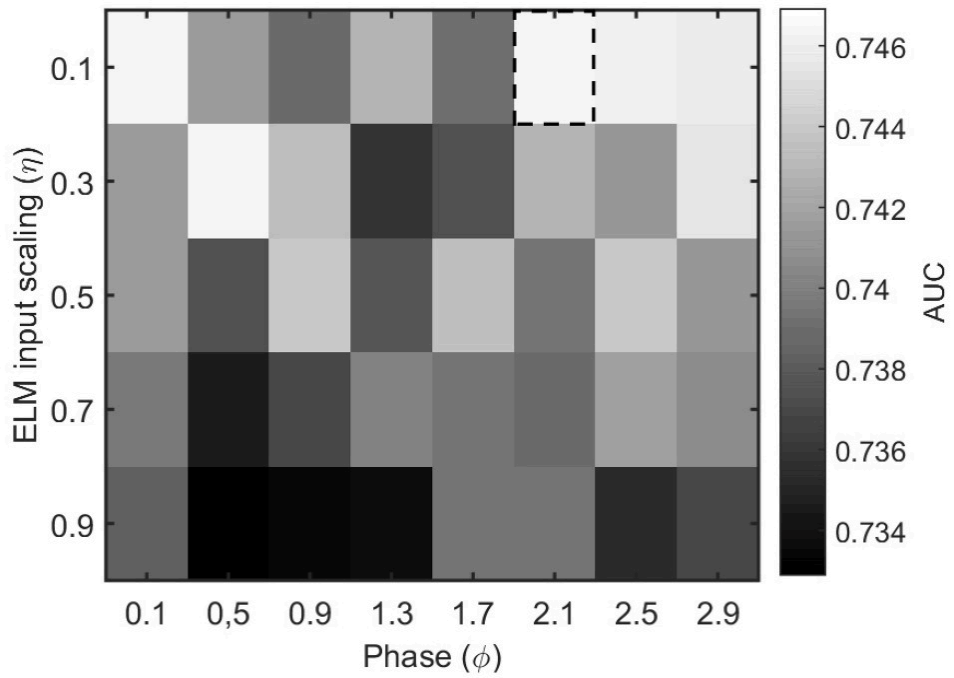
Performance evaluation (AUC) for the classification of healthy and epileptic subjects as a function of the parameters η (input scaling) and ϕ (phase) of the ELM algorithm. The chosen parameter values are indicated by a dashed rectangular box.

The other parameters of the ELM algorithm described in Section 2.4 that need to be tuned are the input scaling η and the phase ϕ. To do so, we test the accuracy of a binary classifier to identify the condition of epilepsy in the MEG segments as a function of the algorithm parameters. Figure 4 illustrates the results for a η and ϕ parameter scan. From the figure, it is clear that the parameter values η = 0.1 and ϕ = 2.1 offer a good classification accuracy and will be kept fixed throughout the rest of the manuscript.

## 3. Results

**Figure 5:**
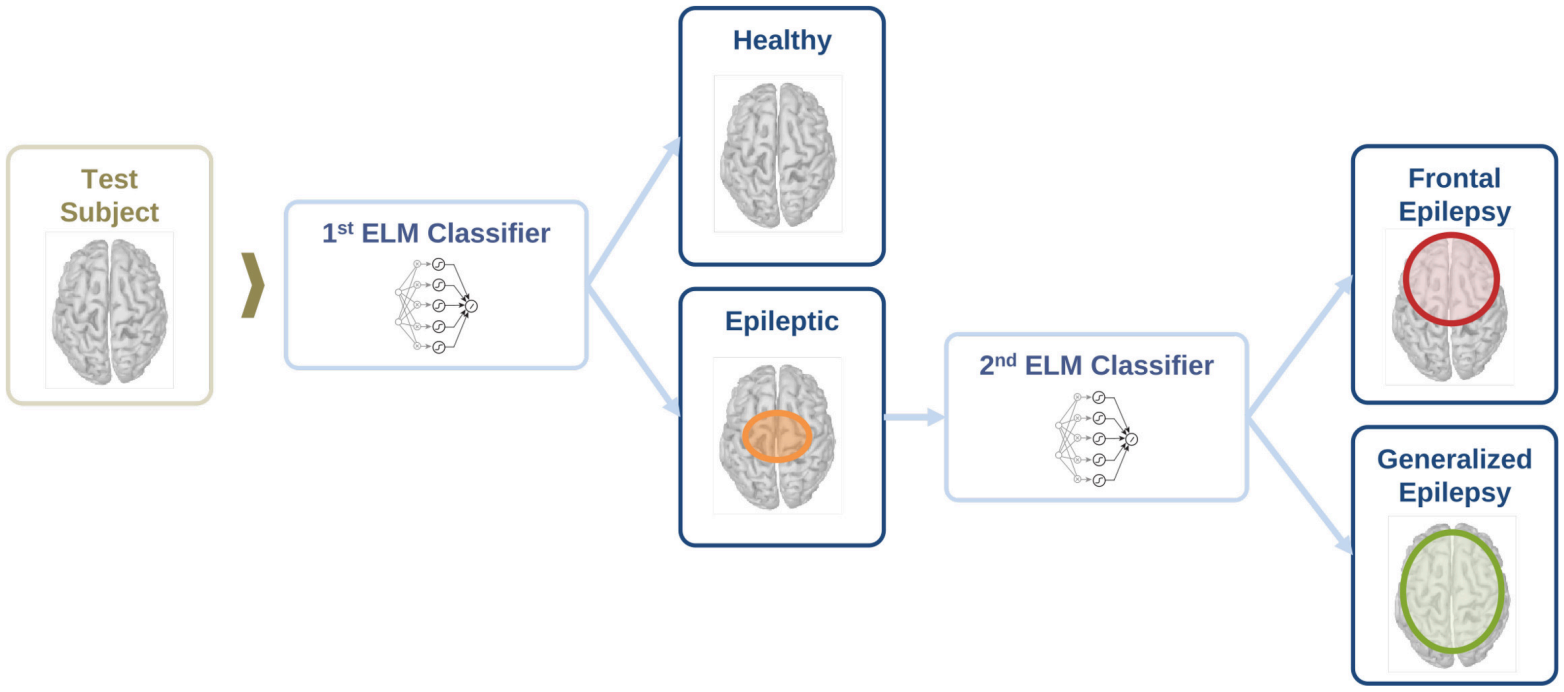
Schematic representation of the information flow to evaluate the condition of a test subject with two sequential binary classifiers.

In this section, we evaluate the feasibility to distinguish between healthy and epileptic subjects from MEG resting activity. Our final goal is to find a procedure to identify the three different conditions in our test group, namely healthy, generalized, and focal. To this end, we design a two-stage procedure to identify the three different conditions. This two-stage classification procedure is illustrated in Figure 5, with an ELM classifier in each stage that decides over two conditions (binary classifier). First, we train the system to automatically distinguish between healthy and epileptic subjects, without specifying the type of epilepsy. Second, we train another ELM classifier to detect generalized and focal traits within the whole group of epileptic subjects. Finally, we test the two-stage classifier designed to identify the three conditions (healthy, generalized, and focal). For all cases, we always report the error in the testing phase.

For each binary classifier in Figure 5, we evaluate the classification performance for the total and relative PSD, and the two phase synchronization measures described in Section 2.3 (i.e., PLV and PLI). The results for each binary classifier are reported in Sections 3.1 and 3.2, respectively, while the final results for the two-stage classification are reported in Section 3.3.

### 3.1. Classification between healthy and epileptic subjects

**Figure 6:**
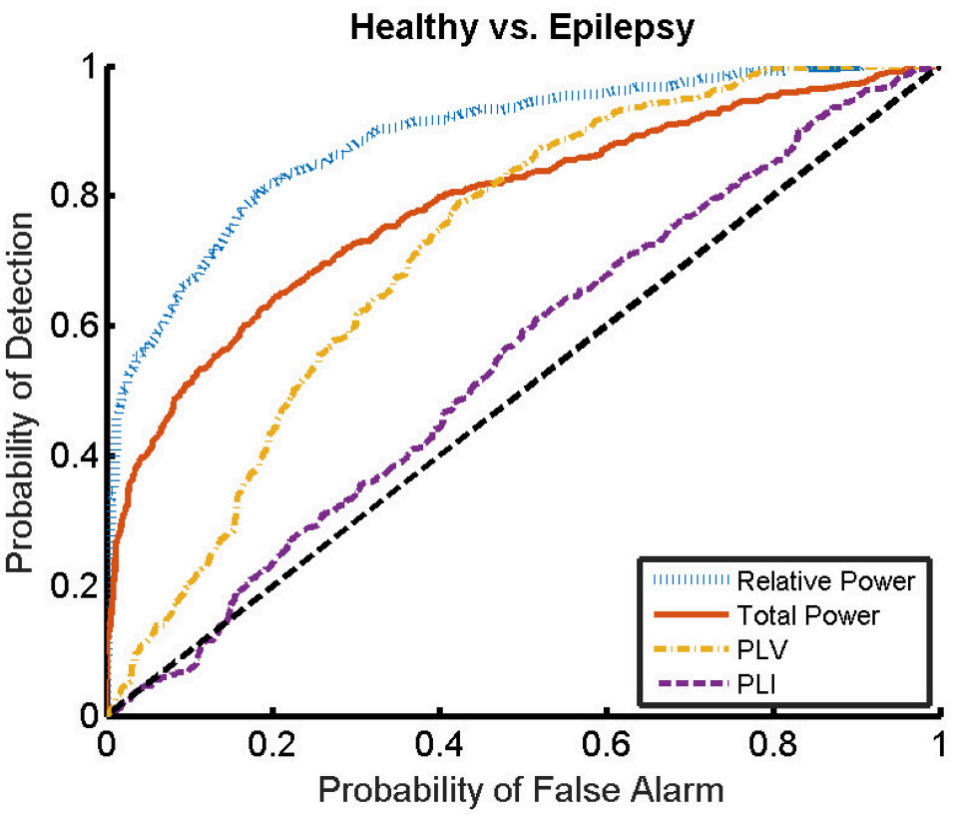
ROC curves for the detection of the presence of epilepsy condition for the relative and total PSD, PLV, and PLI. The diagonal dashed line indicates results equal to chance.

Here, we present the results for the first stage of the automated classifier, trained to distinguish between MEG segments of healthy and epileptic subjects (without specifying the type of epilepsy). We train an ELM independently for each feature to determine the best discriminant feature between healthy and epileptic subjects, each time taking only one of the calculated features as input. Figure 6 shows the ROC curves obtained for the identification of the MEG segments of epileptic subjects for each feature. We find that the best results are obtained when we use the relative PSD as input to the ELM classifier. In turn, the total PSD also performs better than the phase synchronization measures. Finally, PLV is superior to PLI, which yields classification values close to chance.

The ROC curves of Figure 6 illustrate the algorithm performance for the classification of segments. It is, however, also interesting to discuss the results in terms of subjects. To this end, we check the condition (healthy or epileptic) of the corresponding segments of each subject and assign a condition to the subject following a majority rule vote over his/her segments (see Section 2.6 for details). We do it only for the relative PSD, as it gives the best results at the level of segments. The test performance of the classifier at the subject level is summarized in the confusion matrix shown in Table 3. This confusion matrix has an accuracy of 0.9, a specificity of 0.86, and a sensitivity of 0.93. These results show that it is possible to identify epileptic subjects from interictal MEG resting activity with high accuracy using an ELM classifier. In the test phase of the cross-validation procedure, the ELM classification algorithm is able to properly classify 38 out of the 42 subjects. The nonlinear transformation carried out by the ELM is essential to obtain these results, as performing linear regression over the original input features, without applying the ELM algorithm (results not shown), yields classification accuracies close to chance.

**Table 3:** Test confusion matrix of the first stage classifier that is trained to identify healthy vs. epileptic subjects, taking the relative PSD as input to the binary classifier.

		**Prediction**		**Total**
		**Healthy**	**Epilepsy**	
True condition	Healthy	12	2	14
	Epilepsy	2	26	28
	Total	14	28	42

*These results are obtained using the threshold with a probability of detection of 0.8 at the level of segments in the ROC curve presented in Figure 6*.

### 3.2. Within-group classification of the epileptic subjects

We now evaluate which features obtained from the MEG time-series are able to best distinguish between generalized and focal traits. To this end, we train a classifier with only the epileptic subjects. We find that the relative PSD again outperforms the other features for the identification of the epilepsy type, while the PLV is the synchronization measure with the best performance. The overall classification accuracy is, however, low even for the relative PSD. In order to try improving the classification of epilepsy types, we evaluate the performance of the ELM classifier when we restrict the frequency content of the features to specific frequency bands (Niso et al., 2015). In particular, we focus on the theta (4–8 Hz), alpha (8–14 Hz), beta 1 (14–20 Hz), beta 2 (20–28 Hz), and low gamma (28–40 Hz) bands. We find that the relative PSD, using only the beta 1 frequency band, helps best to distinguish between generalized and focal epilepsy. This means that the ELM algorithm classifies better the types of epilepsy if the features are restricted to frequency bands before automated classification. We then search if there are pair-wise combinations of (frequency restricted) features that can further improve the classification. The best classification performance, as measured by the AUC (Table 4), is obtained by using a suitable pair-wise combination of the relative PSD (beta 1) and PLV (beta 2) of the frequency restricted features. The choice of pair-wise combinations is motivated by the hypothesis that these two different features contain complementary information of the underlying data. Indeed, PSD reflects the local activation of each sensor data, whereas the average PLV reflects the statistical relationship between the MEG activity in this sensor and that in the rest of the sensors. Namely, the former is an estimation of local activity, whereas the latter estimates changes in global connectivity.

**Table 4:** Test results for the AUC of the classifiers trained to identify generalized vs. focal epilepsy based on pair-wise combinations of the relative PSD (Rel) and the PLV, restricted to given frequency bands.

	**Theta (Rel)**	**Alpha (Rel)**	**Beta 1 (Rel)**	**Beta 2 (Rel)**	**Gamma (Rel)**
Theta (PLV)	0.5037	0.4955	0.6844	0.6212	0.6860
Alpha (PLV)	0.4417	0.4734	0.6730	0.6191	0.7058
Beta 1 (PLV)	0.4378	0.5433	0.6670	0.5722	0.5531
Beta 2 (PLV)	0.5380	0.5975	**0.7440**	0.6565	0.6328
Gamma (PLV)	0.5680	0.6246	0.7033	0.5764	0.5407

*The largest AUC value is highlighted in bold*.

**Figure 7:**
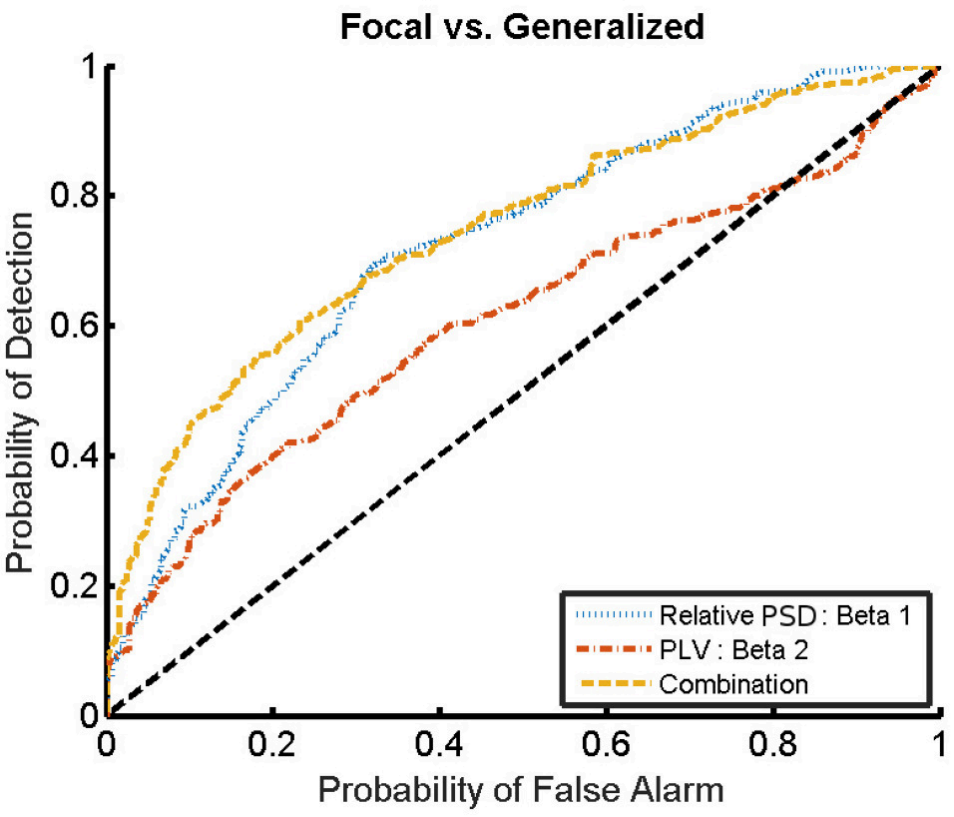
ROC curves for the detection of the epilepsy type for the relative PSD restricted to the beta 1 band, the PLV restricted to the beta 2 band, and the combination of both. The diagonal dashed line indicates results equal to chance.

For the combination yielding the highest AUC in Table 4 (beta 1 band of the relative PSD and beta 2 band of the PLV), the corresponding ROC curve for the MEG segments is presented in Figure 7. In addition to the ROC curve for the optimum combination of input features, we present the ROC curves for these input features considered individually. By comparing these ROC curves, it is apparent that the AUC of this combination is higher than the AUC for the beta 1 band of the relative PSD, alone.

Similarly to the procedure described in Section 3.1, the test results at the subject level for the classifier trained to identify the type of epilepsy are presented in Table 5. These results correspond to the optimal combination detected above of the beta 1 relative PSD and beta 2 PLV. The accuracy of this binary classifier is 0.93 (specificity of 0.86 and sensitivity of 1). We find that, in the test phase, the classifier is able to properly identify the type of epilepsy for 26 out of the 28 epileptic subjects studied.

**Table 5:** Test confusion matrix of the ELM classifier trained to identify generalized vs. focal epilepsy.

		**Prediction**		**Total**
		**Generalized**	**Focal**	
True condition	Generalized	12	2	14
	Focal	0	14	14
	Total	12	16	28

*The ELM inputs are the beta 1 band of the relative PSD and the beta 2 band of the PLV. These results are obtained using the threshold that gives a probability of detection of 0.6 at the level of segments in the ROC curve presented in Figure 7*.

### 3.3. Full classification of subjects

As reported in previous sections, it is possible to distinguish between epileptic and healthy subjects (stage 1) and epileptic subjects with general or focal epilepsy (stage 2) with high accuracy. In this section, we report the test results for the two-stage classification procedure illustrated in Figure 5. This procedure allows identification of the three different conditions (healthy, generalized, and focal). The complete classification procedure contains two binary ELM classifiers. Thus, each test subject is first classified as healthy or epileptic. In the latter, a second classifier further discriminates the type of epilepsy.

We present the confusion matrix for the full classifier in Table 6. As input features, we choose the relative PSD for the first stage and the combination of beta 1 relative PSD and beta 2 PLV for the second stage. In the test phase of the final classifier, 36 out of the 42 subjects are properly classified. The accuracy of the full classifier for the three conditions is 0.86, with a sensitivity of 0.88, and a specificity of 0.86.

**Table 6:** Test confusion matrix of the final two-stage classification algorithm.

		**Prediction**			**Total**
		**Healthy**	**Generalized**	**Focal**	
Truth	Healthy	12	0	2	14
	Generalized	1	11	2	14
	Focal	1	0	13	14
	Total	14	11	17	42

## 4. Discussion

From the neuroscientific point of view, the prediction of epileptic activity from interictal signals is a challenging task. Here, we compute several features from interictal resting state MEG activity and evaluate if they can be used for the identification of epilepsy. Our analysis suggests that a simple feature such as the relative PSD outperforms the other features considered here in the correct identification between epileptic and healthy subjects. This is not completely surprising since the MEG technique seems to produce distinct signals in healthy and epileptic subjects even during interictal resting state without any epileptic discharges (Englot et al., 2015; Niso et al., 2015). In our case, the power distribution in the whole frequency spectrum (relative PSD), rather than the total power or the phase synchronization indicators (PLV and PLI), gives the best results to distinguish these two groups automatically and with high classification accuracy. Importantly, from the practical point of view, the relative PSD is computationally less expensive and faster to calculate than both the PLV and PLI.

The discrimination between the two different types of epilepsy considered here turned out to be a more challenging task. Indeed, neither local (PSD) nor global activation patterns (PLV) alone are able to accurately distinguish between idiopathic generalized epilepsy and frontal focal epilepsy. Rather, for this purpose it is necessary to combine both types of features, restricted to certain bands, to achieve a better-than-chance performance. In this case, the best results are obtained by combining the relative PSD restricted to the beta 1 band and the PLV restricted to the beta 2 band. This result can be interpreted as a proof of concept of the importance of the feature selection process. In this case, the frequency ranges over which the PLV and the relative PSD are restricted to may be specific to the population that we have investigated here. The limitations of the dataset we used precludes the possibility to validate this hypothesis on a larger population, including different pathologies. PSD and PLV characterize in principle different aspects of brain activity, thereby providing complementary information about local activation and global connectivity that can be suitably combined for classification. When the analyzed groups are different in terms of activity, one of the features may be enough for the classification task. If they are similar, however, it is necessary to combine both types of features. We note that there might be prospective physiological mechanisms behind the improvements on the identification of the epilepsy types when using combined measures. Typically, the discrimination between generalized and focal epilepsy in single patients is performed by analyzing spikes and seizures of long EEG recordings (Duffy et al., 1994). Here, we offer a complementary approach that can perform such discrimination even in the absence of spikes and seizures.

It is also interesting to note that the PLI systematically failed to produce accurate classifications between healthy and epileptic groups, even more so between the two epileptic ones. The main difference between PLV and PLI is that the former measures phase synchronization with and without time lag. Zero lag coupling may be physiologically meaningful (Vicente et al., 2008) or it could be the result of the linear mixing, in two different sensors, of the activity of common underlying neural sources (Stam et al., 2007). In contrast to the PLV, the PLI is not sensitive to zero lag coupling and detects only phase lagged connectivities. While this feature makes the results from the PLI easier to interpret, recent results on MEG resting state data (Colclough et al., 2016; Garcés et al., 2016) clearly suggest that it is at the expense of sacrificing reliability. Although, the results from the two aforementioned studies were obtained at the source level, whereas our results are obtained at the sensor level, the main conclusion still remains: both variability within group and between sessions for the same subject for many functional connectivity measures are much higher if one removes zero lag coupling. From our results, it is clear that such variability hinders the use of PLI in the present framework as a reliable biomarker to distinguish among the three groups of subjects considered. This limitation could be overcome by using more sophisticated methods to detect phase-lag synchronization (Vinck et al., 2011). It is also worth noting that a recent work combining anatomical and functional (EEG) information for modeling (Finger et al., 2016) found that a considerable amount of functionally relevant synchrony takes place with near zero or zero-phase lag. This is in line with our findings, and suggest that they may not be a methodological issue but rather a fundamental property of the neurophysiological activity.

In summary, we have demonstrated that an automated classification algorithm is able to identify healthy and epileptic subjects from brain activity measured with MEG in a resting state. The identification of the type of epilepsy within subjects with idiopathic generalized epilepsy or frontal focal epilepsy was also successfully achieved. This is clinically relevant since these two epilepsy groups show similar semiology.

## 5. Outlook

In this manuscript, we identified a combination of features that allows the automated classification of idiopathic generalized epilepsy and frontal focal epilepsy. We do not exclude the possibility that other features could contribute to improve even further such classification and future work will be carried out in this direction. In particular, after the results obtained by combining PSD and PLV from different frequency bands, it would be interesting to study the possible role of cross-frequency coupling in this framework, in line with very recent studies in resting state MEG (Florin et al., 2015; Tewarie et al., 2016). In addition, motivated by the classification improvements using the majority rule, we expect that studies with longer monitoring periods and an increased number of subjects would yield an even better generalization performance of the algorithm.

It is worth noting that the algorithm discussed in Section 2.4 can be implemented in electronic and optoelectronic hardware (Decherchi et al., 2012; Ortín et al., 2015; Soriano et al., 2015b). Even though this manuscript has focused on the analysis of MEG time-series, which are measured by high-cost devices, similar principles can be applied to the analysis of EEG time-series. Therefore, one could think of combining both the measurement of the brain activity and the machine learning algorithm in a single low-cost hardware device. Such a device could allow, for e.g., real-time seizure detection, a problem in which machine learning algorithms based on random mappings have already been validated (Buteneers et al., 2013).

Machine learning algorithms are becoming ubiquitous in the analysis of biomedical data. Attempts to achieve a convergence between machine learning concepts and neuroscience are currently an active topic of research (Marblestone et al., 2016; Xia et al., in press). Here, we contributed to this research field by identifying distinctive features of epileptic and healthy subjects.

## Author contributions

MS, CM, and EP designed the study. MS, JC, and SO analyzed the data using ELM. SC and MG diagnosed and selected the participants in the study. GN recorded, preprocessed, and performed the MEG data analysis. MS drafted the manuscript. All authors critically revised the manuscript and approved the final version.

### Conflict of interest statement

The authors declare that the research was conducted in the absence of any commercial or financial relationships that could be construed as a potential conflict of interest.
